# Clearance of p16‐positive cardiac cells improves age‐related cardiac remodeling in mice

**DOI:** 10.1113/EP093551

**Published:** 2026-09-14

**Authors:** Mozhdeh Mehdizadeh, Martin Mackasey, Kimia Gharagozloo, Martin Aguilar, Patrice Naud, Allan Ochs, Nhung Vuong‐Robillard, Eric Thorin, Gerardo Ferbeyre, Jean‐Claude Tardif, Martin G. Sirois, Jean Francois Tanguay, Stanley Nattel

**Affiliations:** ^1^ Research Center Montreal Heart Institute Université de Montréal Montreal Canada; ^2^ Department of Pharmacology and Therapeutics McGill University Montreal Canada; ^3^ Department of Medicine Université de Montréal Montreal Canada; ^4^ Department of Biochemistry Université de Montréal and CRCHUM Montreal Canada; ^5^ Department of Surgery Université de Montréal Montreal Canada; ^6^ Department of Pharmacology and Physiology Faculty of Medicine Université de Montréal Montreal Canada; ^7^ Institute of Pharmacology West German Heart and Vascular Center University Duisburg‐Essen Essen Germany

**Keywords:** cardiac fibrosis, cellular senescence, diastolic dysfunction

## Abstract

Senescent cells are characterized by expression of markers like p16 and secretion of profibrotic and proinflammatory factors. The role of cellular senescence in age‐related cardiac remodeling and dysfunction is incompletely understood. This study aimed to: (i) evaluate the effect of p16‐ positive cell clearance on cardiac function and structure in aging mice, and (ii) assess the role of different cardiac cell‐types in the response. Hypertrophy markers, ion channels and calcium handling protein gene expression. Statistical analysis for all panels: one‐way ANOVA followed by Tukey’s test, significance level P<0.05 (N=6 for each group). Each point represents results from one mouse; bars and horizontal lines are means and SD. NK‐ATTAC mice, permitting targeted clearance of p16‐positive cells upon exposure to the dimerizing agent AP20187 (AP), were treated with AP or vehicle from 12 to 18 months of age. Cardiac function and structure were assessed with echocardiography, hemodynamics with a Millar catheter. p16‐positive cells in various cardiac cell populations were analyzed with Fluorescence‐Activated Cell Sorting (FACS) and immunofluorescence imaging. Echocardiography revealed significant attenuation of aging‐associated increases in left ventricular mass to diameter at end‐diastole (LVDd) and anterior wall thickness at end diastole (LVAWTd) in Aged‐AP mice versus Aged‐Vehicle. Diastolic dysfunction in vehicle mice normalized with AP treatment. FACS results indicated clearance of p16‐positive fibroblasts with AP. Immunofluorescence imaging indicated reduced p16‐positive fibroblasts and cardiomyocytes with AP, implicating them in the effects of p16‐positive cell clearance on age‐related cardiac remodeling. Exposure of cardiomyocytes to senescent fibroblast products led to upregulation of hypertrophy markers, pointing to paracrine effects on cardiomyocytes. This study highlights the potential contribution of senescent fibroblasts and cardiomyocytes to age‐related cardiac remodeling. Modulating senescence might provide a new approach to age‐related cardiac diseases like heart failure.

## Abstract (highlights)

- **What is the central question of this study?**
  Does clearing of p16‐positive cells in the aging heart attenuate age‐related cardiac dysfunction? Which cell‐types are involved?
- **What is the main finding and its importance?**
  Selective clearance of cardiac cells with a p16‐targeted probe reduces age‐related signs of diastolic dysfunction, cellular hypertrophy, and fibrosis. These effects appear to be due to the targeting of senescent fibroblasts and cardiomyocytes. These findings provide information that may help to prevent the major adverse cardiac effects of aging.

## 1 INTRODUCTION

Age is the leading risk factor for cardiac disease (Gjesdal et al., 2011; Steenman & Lande, 2017; Strait & Lakatta, 2012). The prevalence of heart failure (HF) rises from < 2% in adults younger than 60 years of age to 14.7% of men and 12.8% of women over 80 (Sessions & Engler, 2016; Shih et al., 2011). The underlying changes include impaired left ventricular (LV) relaxation, diastolic dysfunction, LV hypertrophy (LVH), and fibrosis (Shih et al., 2011; Steenman & Lande, 2017). The mechanisms through which aging makes the heart more susceptible to HF remain poorly understood.

The molecular mechanisms underlying cardiac aging are multifactorial and interactive (Li et al., 2020). Cellular senescence is a stress‐ and age‐related response that causes cell‐cycle arrest in dividing cells and is regulated through two key pathways: (i) p16–retinoblastoma protein and (ii) p53–p21 (Di Micco et al., 2021; Mehdizadeh et al., 2022). Senescent cells are resistant to apoptosis and secrete proinflammatory cytokines, growth factors, and matrix remodeling proteases, manifesting a “senescence‐associated secretory phenotype” (SASP). Although cellular senescence was initially considered unique to dividing cells, more recent research points to the occurrence of senescence in non‐dividing cells like cardiomyocytes (Anderson et al., 2019).

Recent work suggests the therapeutic potential of targeting senescence in the prevention and/or treatment of age‐related cardiac disease. Baker et al. showed that the clearance of p16‐positive senescent cells in aged mice reduces cardiomyocyte size (Baker et al., 2016). Similarly, Anderson et al. showed that the clearance of senescent cells with the senolytic drug navitoclax or in the p16‐targeting INK‐ATTAC transgenic mouse model results in reduced cardiomyocyte size and fibrosis (Anderson et al., 2019). To our knowledge, no data are available in the literature regarding the effects of senescent‐cell clearance on LV hemodynamic parameters, systolic and diastolic function, and electrophysiological properties. Furthermore, the cell‐specificity underlying the effects of cellular senescence on age‐related cardiac remodeling is often ignored.

Here, we tested the hypothesis that cells expressing the senescence‐marker p16 contribute to age‐related cardiac‐function remodeling. To test our hypothesis, we studied aged INK‐ATTAC mice treated with the dimerizing agent AP20187 (AP), which induces apoptotic cell‐death in p16‐positive cells, or with a matching vehicle (Baker et al., 2016). Our specific goals were: (1) to evaluate the effect of p16‐positive cell clearance on LVH and fibrosis, as well as LV systolic and diastolic function in aged mice; (2) to study the effect of p16‐positive cell clearance on hemodynamic and conduction indices; (3) to evaluate the role of different p16‐positive cardiac cell‐types in age‐related cardiac remodeling; (4) to evaluate potential paracrine effects of senescent fibroblasts.

## 2 METHODS

### 2.1 Ethical approval

All animal procedures were reviewed and approved by the Institutional Animal Care Committee of the Montreal Heart Institute (protocol number: 2019‐47‐04). All experiments were conducted in accordance with the guidelines of the Canadian Council on Animal Care (CCAC). Animals were maintained and cared for in accredited animal care facilities in accordance with Experimental Physiology's policies on animal experimentation. All procedures were performed by trained personnel, and every effort was made to minimize animal suffering. Animals were monitored regularly throughout the study, and humane endpoints were applied when necessary to reduce pain and distress. Animal welfare monitoring, including body weight measurements, was conducted at least once per week, and more frequently for animals undergoing treatment. Analgesia was not administered routinely, as no surgical procedures were performed. However, when animals exhibited signs of pain or distress, ketoprofen was administered subcutaneously at 20 mg/kg once daily. Dermatological conditions were managed with topical Theraderm cream and/or application of green clay to affected areas once daily (SID) for 5 days. In cases of early lesion development, maropitant citrate (Cerenia) was administered subcutaneously at 1 mg/kg once daily for 5 days. Animals had ad libitum access to food and water and were maintained under standard light–dark cycle conditions in accordance with CCAC guidelines. Appropriate anesthesia was achieved using inhaled 2% isoflurane in oxygen. Depth of anesthesia was assessed by absence of the pedal withdrawal (toe pinch) reflex and lack of response to tactile stimulation. Anaesthesia was maintained by adjusting isoflurane concentration as needed and monitored throughout the procedure by observing respiratory rate and pattern. Mice were euthanized by cardiac excision under anesthesia. The authors confirm that they understand the ethical principles under which the journal operates and that this study complies with the journal's animal ethics checklist.

### 2.2 Animal model

We used the male INK‐ATTAC mouse model to eliminate p16‐positive cells by leveraging a genetic approach based on the AP‐induced dimerization of a membrane‐bound myristoylated FK506‐binding‐protein–caspase 8 (FKBP–Casp8) fusion protein with expression driven by a modified p16‐gene promoter (Baker et al., 2016). Caspase‐8 requires dimerization for activation, which specifically occurs within cells that overexpress p16. After the fusion proteins dimerize, activated caspase initiates apoptotic cell‐death and clearance of cells that express p16. Authorization was obtained from Unity Biosciences (California, USA) to acquire INK‐ATTAC transgenic mice by sourcing cryopreserved sperm from Jackson Laboratory. The sperm samples were then transported to the CHUM Research Centre in Montreal, where they were rederived into live mice through in vitro fertilization (IVF). This process enabled us to establish a breeding colony for subsequent experiments.

### 2.3 Animal treatment

**FIGURE 1:**
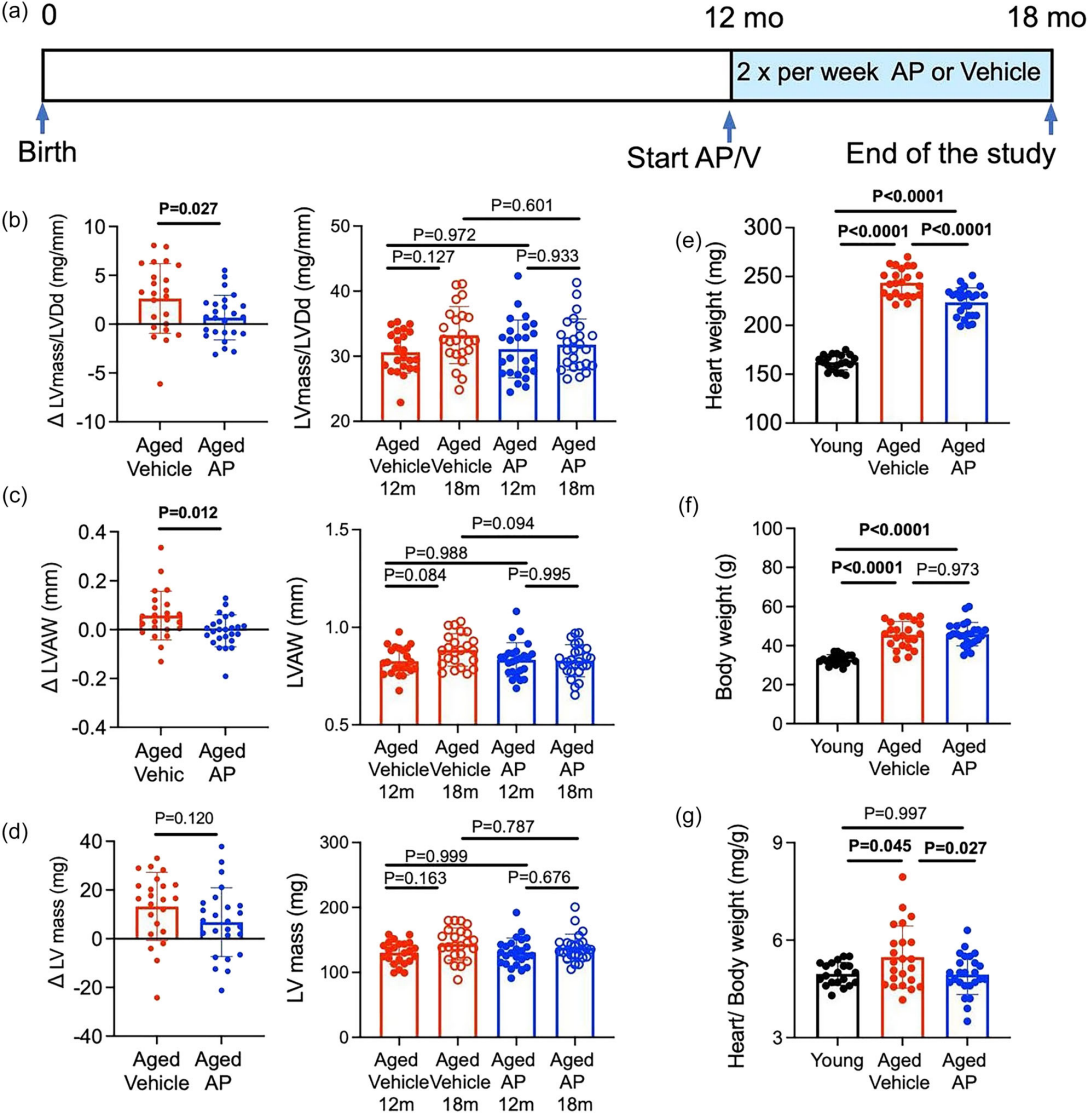
Study design and treatment‐associated changes in structural indicators of hypertrophy in aged INK‐ATTAC mice treated with vehicle or AP. (a) Study design: INK‐ATTAC mice were treated twice‐weekly with vehicle or AP, from 12 months until 18 months of age. (b) Changes in left ventricular (LV) mass/end‐diastole dimension (LVDd) and absolute values at 12 and 18 months. (c) Changes in LV anterior wall thickness (LVAWT) and absolute values at 12 and 18 months. (d) Changes in LV mass and absolute values at 12 and 18 months. (e) Heart weight. (f) Body weight. (g) Heart weight normalized to body weight (For all analyses in b–g, Young mice: *n* = 20; Aged mice: Vehicle‐treated: *n* = 23; AP‐treated: *n* = 25). Statistical analysis for changes in echocardiogram parameters: unpaired *t*‐test; for the rest of the analyses: one‐way ANOVA followed by Tukey's test. Echocardiographic results are reported as absolute values and changes occurring from 12 to 18 months during treatment with AP (Aged‐AP) or Vehicle (Aged‐Vehicle). Each point represents results from one mouse; bars and horizontal lines are means and SD.

INK‐ATTAC mice were randomly allocated to the following groups: Young (4 months, without treatment), Aged‐AP (18 months, with 6‐month AP treatment), and Aged‐Vehicle (18 months, with 6‐month vehicle treatment). Investigators were blinded to the identity of AP versus vehicle treated mice. AP (2 µg/g body weight) or vehicle (4%‐ethanol, 10% PEG400, 86% Tween 2% in water) treatment for aged groups began when the mice were 12 months old (Figure 1a). The mice received these treatments by intraperitoneal injection two times a week for six months, based on prior work (Baker et al., 2016).

### 2.4 Drugs and chemicals

**Table:** 

Name	Category	Supplier	Identifier (Catalog #)	Application
AP20187	Drug	Takara Bio (Shiga, Japan)	635069	Dimerizing agent used to induce apoptosis in p16+ cells of INK‐ATTAC model
Taurine	Chemical	Bioshop Canada (Burlington, ON)	TAU303	Component of experimental solutions
Blebbistatin		Toronto Research Chemical (North York, ON)	B592490‐10	Inhibits cardiac contraction during optical mapping (reduces motion artifacts)
RH‐237	Dye	Invitrogen (Waltham, MA)	S1109	Voltage‐sensitive dye for optical mapping
Alexa Fluor 647 Rat Anti‐Mouse CD31	FACS Antibody	BD Bioscience (Mississauga, ON)	563608	Endothelial cell marker for FACS
BV421 Rat Anti‐Mouse CD45		BD Bioscience (Mississauga, ON)	563890	Immune cell marker for FACS
p16	Primary Antibody	Abcam (Cambridge, UK)	ab54210	Senescence marker
α‐Smooth Muscle Actin (α‐SMA)		Abcam (Cambridge, UK)	ab5694	Myofibroblast marker
Troponin I		Abcam (Cambridge, UK)	ab47003	Cardiomyocyte marker
Vimentin		Cell Signaling (Danvers, MA)	3932S	Fibroblast marker
CD31		R&D Systems (Minneapolis, MN)	AF3628‐SP	Endothelial cell marker
4’,6‐diamidino‐2‐phenylindole (DAPI)	Stain	Invitrogen (Waltham, MA)	—	Nuclear stain
Wheat Germ Agglutinin (WGA)		Invitrogen (Waltham, MA)	W32466	Cell membrane staining
555 Donkey anti‐mouse	Secondary Antibody	Invitrogen (Waltham, MA)	A31570	Immunofluorescence detection
488 Donkey anti‐rabbit		Invitrogen (Waltham, MA)	A21206	Immunofluorescence detection
488 Donkey anti‐goat		Invitrogen (Waltham, MA)	A11055	Immunofluorescence detection
FcR blocker (purified anti‐mouse CD16/32)	Reagent	BioLegend (San Diego, CA)	101302	Prevents nonspecific antibody binding in FACS
Fixable Viability Stain 780 (FVS)		BD Bioscience (Mississauga, ON)	565388	Distinguishes live/dead cells in FACS
12 mm Transwell® with 0.4 µm Pore Polycarbonate Membrane Insert	Consumable	Corning Inc (Corning, NY)	29442‐086	Coculture system for paracrine studies
Hydroxyproline assay kit	Assay Kit	Sigma‐Aldrich (Missouri, USA)	MAK008‐1KT	Quantification of collagen content

### 2.5 Echocardiography

Echocardiographic studies were performed as previously described (Moreira et al., 2020). Briefly, mice were anesthetized with 2% isoflurane and kept on a heated platform to maintain the body temperature around 37°C. Cardiac function and structure were assessed by transthoracic echocardiography using an i13L probe (10–14 MHz) and Vivid 7 Dimension ultrasound system (GE Healthcare Ultrasound, Horten, Norway). LV anterior‐wall thickness at the end of diastole (LVAWTd), LV dimension at end cardiac diastole (LVDd) and systole (LVDs) were measured by M‐mode echocardiography (M‐mode). LV fractional shortening and ejection fraction were obtained with the formulas available within the Vivid 7 system. LV transmitral filling velocities in early (E) and atrial (A) filling‐phases were measured with pulsed wave Doppler (PW). LV mass was calculated using a previously described formula (Liao et al., 2002). Left atrial dimension at end cardiac systole and diastole (LADs, LADd) were measured by M‐mode. The average of 3 consecutive cardiac cycles was used for each measurement. Throughout the experiments, the operator was blinded to group/treatment assignment.

### 2.6 Histology

Formalin‐fixed, paraffin‐embedded samples were cut at 6‐µm thickness. Image Pro Premier 9.3 Software (Media Cybernetics, Rockville, MD) was used to quantify fibrosis on Masson's Trichrome‐stained images.

Whole LV sections were double‐stained for p16 and cell‐type–specific markers, scanned (Aperio VERSA Brightfield Scanner, Leica Biosystems, Nussloch, Germany), and the entire scanned image analyzed using an automated image‐analysis pipeline (Visiomorph, Visiopharm, Horsholm, Denmark). All analyses were performed blinded to mouse‐group identity using consistent thresholds across all samples.

For cardiomyocyte analysis, cell boundaries were defined using wheat germ agglutinin (WGA) staining, which outlines cell membranes and enables segmentation of individual cells. Cardiomyocyte regions of interest (ROIs) were identified based on troponin I (TnI) positivity. Nuclei were detected using DAPI staining, and nuclei located within TnI‐positive ROIs were classified as cardiomyocyte nuclei. p16^+^ cardiomyocyte nuclei were identified based on colocalization of p16 signal with DAPI within these ROIs. Nuclei outside TnI‐positive regions were excluded from cardiomyocyte analysis.

For non‐cardiomyocyte populations, nuclei were detected using DAPI staining and assigned to specific cell types using a spatial association approach, as cell‐type markers were not nuclear. Endothelial nuclei were defined as DAPI‐positive nuclei located within 2 µm of CD31‐positive signal. Fibroblast‐associated nuclei were defined as DAPI‐positive nuclei located within 2 µm of vimentin‐positive signal. Myofibroblast‐associated nuclei were defined as DAPI‐positive nuclei located within 2 µm of α‐smooth muscle actin (α‐SMA)‐positive signal.

### 2.7 Hydroxyproline assay

Total collagen content in the LV was determined by hydroxyproline assay as described previously (Jansen et al., 2019). The results were expressed as µg hydroxyproline/mg dry tissue.

### 2.8 Cardiac cell isolation

To isolate cardiomyocytes and non‐myocyte cardiac cells (including fibroblasts, myofibroblasts, immune cells, and endothelial cells), we performed Langendorff perfusion of cannulated mouse hearts with the previously described method (O'Connell et al., 2007). The mice were injected with 10,000 IU heparin (in 0.2 mL 0.9%‐saline/mouse) for anticoagulation, anesthetized with 2% isoflurane, and euthanized by cardiac excision. The heart was cannulated using fine‐tip forceps to slide the aorta onto the cannula so that the tip of the cannula was just above the aortic valve and perfused with perfusion buffer to wash out the remaining blood in the heart, followed by a 16‐min perfusion with the same solution containing collagenase II (2.4 mg/mL; first 6‐min period without calcium and the next 10‐min interval with the same solution containing 40 µM CaCl_2_). Tissues were minced and cellular dissociation was completed by gentle trituration. The cell suspension was first passed through a 500‐µm filter and centrifuged at 60 g to obtain cardiomyocytes (the pellet). Then, the supernatant was passed through a 20 µm filter and centrifuged at 500 g to obtain non‐myocyte cardiac cells.

### 2.9 Fluorescence activated cell sorting (FACS)

The isolated non‐myocyte cardiac cells were resuspended in the sorting buffer (PBS (Ca^2+^/Mg^2+^ free), 20‐mM HEPES, pH 7.4, 5‐mM EDTA, 0.5% BSA) and stained with FVS (to distinguish dead cells), then blocked for Fc receptors with Fc‐blockers. Cells were then exposed to antibodies to cell‐surface markers of endothelial cells (Alexa Fluor 647 Rat Anti‐Mouse CD31) or immune cells (BV421 Rat Anti‐Mouse CD45). The double negative population was considered to represent fibroblasts; this assumption was confirmed by assay for the expression of cell‐type selective genes. As the INK‐ATTAC transgene contains a green fluorescent protein (GFP) sequence, p16‐positive cells were identified based on GFP‐positivity. Cells were washed, resuspended in the sorting buffer, and sorted with a FACS Aria Fusion (BD Biosciences) cell‐sorter into four different populations: double negative (fibroblasts) GFP^+^, double negative (fibroblasts) GFP^−^, CD31^+^(endothelial cells), and CD45^+^ (Immune cells). Cells were then used for qPCR experiments to quantify specific genes of interest. The percentage of p16‐positive cells in all cell populations were determined based on the GFP signal. The data were analyzed with FlowJo V10.7.1 (BD Biosciences). Prior to sorting, we performed compensation to correct for spectral overlap between the fluorophores used in the experiment, by running single‐stained compensation controls using beads conjugated with each specific antibody. These controls were essential for accurately setting the gates and ensuring that the fluorescence signals from different channels were correctly distinguished. The compensation matrix was then applied in FlowJo V10.7.1 (BD Biosciences) to adjust the data and allow for precise identification and sorting of the cell populations

### 2.10 Hemodynamic study

The mice were anesthetized with 2%‐isoflurane on a temperature‐regulated operating table that maintained the body temperature at 37°C. Through a cervical incision, the right carotid artery was isolated. With a surgical microscope (Leitz Wild M650, Wild Surgical Microscopes, Heerbrugg, Switzerland), a Millar catheter (SPR 671, TX, USA) was inserted and advanced into the ascending aorta, and subsequently into the LV. Transmitted pressure and a surface ECG were recorded and analyzed off‐line with Iox2 software (v.2.8.0.13, EMKA Technologies, Paris, France).

### 2.11 Ex vivo optical mapping

The heart was excised and perfused via the aorta with Krebs solution at 4 mL/min and 37°C, aerated with 95% O_2_/5% CO_2_ to maintain pH at 7.4. After 20 min of stabilization, Krebs solution containing blebbistatin (10 µM) was used to suppress mechanical contraction and prevent motion artifacts. A 750‐µL bolus of 100 µM of the voltage‐sensitive dye RH‐237 was then injected in ∼200 µL increments over 5 min directly into the perfusion line. Experiments were performed in sinus‐rhythm or paced (8‐Hz) RH‐237‐loaded whole hearts illuminated with light from an X‐Cite Xylis Broad Spectrum LED Illumination System (model No: XT720L, Excelitas Technologies, ON, Canada) and filtered with a 520/535‐nm excitation filter. Emitted fluorescence was separated by a dichroic mirror (560 nm cut‐off) and filtered by a 695 long‐pass emission filter. Recordings were captured using a high‐speed CMOS camera (MiCAM03‐N256, SciMedia, Costa Mesa, CA). We mapped conduction and measured action‐potential duration (APD) to 50% (APD50), 70% (APD70), and 90% (APD90) in the LV. Data were captured at a frame‐rate of 1818 or 2914 frames/s using BrainVision software (BrainVision Inc., Morrisville, NC). The spatial resolution was 0.02643 mm/px. Magnification was constant (1.6X) in all experiments, and no pixel binning was used. All recordings were analyzed with custom‐made MatLab software created by Alexander Quinn, Dalhousie University.

### 2.12 qPCR

Total RNA was extracted using the RNeasy Mini Kit. RT‐PCR was performed with Applied Biosystems Thermal Cycler Step One Plus (ThermoFisher Scientific, Waltham, MA). SYBR green primers used for p16 (Fwd: CCCAACGCCCCGAACT, Rev: GCAGAAGAGCTGCTACGTGAA) p21 (Fwd: GGCAGACCAGCCTGACAGAT, Rev: TTCAGGGTTTTCTCTTGCAGAAG), myosin heavy chain 6 (*Myh6*) (Fwd: CCAACACCAACCTGTCCAAGT, Rev: AGAGGTTATTCCTCGTCGTGCAT) and Myh7 (Fwd: CTCAAGCTGCTCAGCAATCTATTT, Rev: GGAGCGCAAGTTTGTCATAAGT), Interleukin‐6 (Il‐6) (Fwd: TCCGGAGAGGAGACTTCACA, Rev: TGCAAGTGCATCATCGTTGT), transforming growth factor beta‐2 (Tgfβ2) (Fwd: TGCCTTCGCCCTCTTTACATT, Rev: AGCGGAAGCTTCGGGATTTA). All mRNA values were normalized to the geometric mean of B2m, Hprt1, and Gapdh. For all analyses, the operator was blinded to group/treatment assignment.

### 2.13 Induction of senescence in cardiac fibroblasts with H_2_O_2_

Primary mouse fibroblasts were isolated via Langendorff perfusion as mentioned above. Fibroblasts were collected from the cell‐suspension by centrifuging at 500 g for 5 min. Fibroblasts were placed in culture using maintenance medium (Dulbecco's Modified Eagle Medium (DMEM), 10% fetal bovine serum, 1% penicillin and streptomycin, and 0.1% amphotericin B). The cells were then washed, and the medium was changed after 2 and 24 h to remove unattached cells. Fibroblasts were cultured for 4 days to reach confluence; they were then trypsinized (passage‐1), counted, and seeded (30,000‐40,000 cells) on each well of 12 well plates. The day after passage‐1, the cells reached almost 60%–70% confluency and were treated with different concentrations of H_2_O_2_, (25, 50, 100, 200, 400, or 800 µM) for two hours, repeated 3 times over two days. The 200 µM concentration was selected for detailed investigation because of its high efficiency in senescence induction, determined by the high level of p16 and p21 with minimal cell death. The senescent fibroblasts were then cocultured with freshly isolated cardiomyocytes using Corning Transwell inserts.

### 2.14 Coculture of senescent fibroblasts and cardiomyocytes

Freshly isolated cardiomyocytes were seeded on laminin‐coated Coring Transwell inserts after calcium reintroduction as described previously (O'Connell et al., 2007). The cardiomyocytes were left to attach for 1 h and then washed. The inserts containing cardiomyocytes were then suspended over the senescent (treated with H_2_O_2_) or control fibroblasts in the 12‐well plate, so that the base of the inserts was within the culture medium on the fibroblasts but did not touch the bottom of the plate. Following this, the co‐cultures were incubated at 37°C and 5% CO_2_ for 48 h before analysis.

### 2.15 Statistical analysis

Statistical analyses were performed using GraphPad Prism 9 (San Diego, CA). For normally distributed data (assessed by Shapiro‐Wilk test), we used Student's *t*‐test (for 2‐group only comparisons) or 1‐way ANOVA (followed by Tukey post‐hoc test). Results are expressed as mean ± Standard deviation (SD), and 2‐tailed *P *< 0.05 was considered statistically significant.

## 3 RESULTS

### 3.1 Effect of p16‐positive cell clearance on cardiac structure and function

**FIGURE 2:**
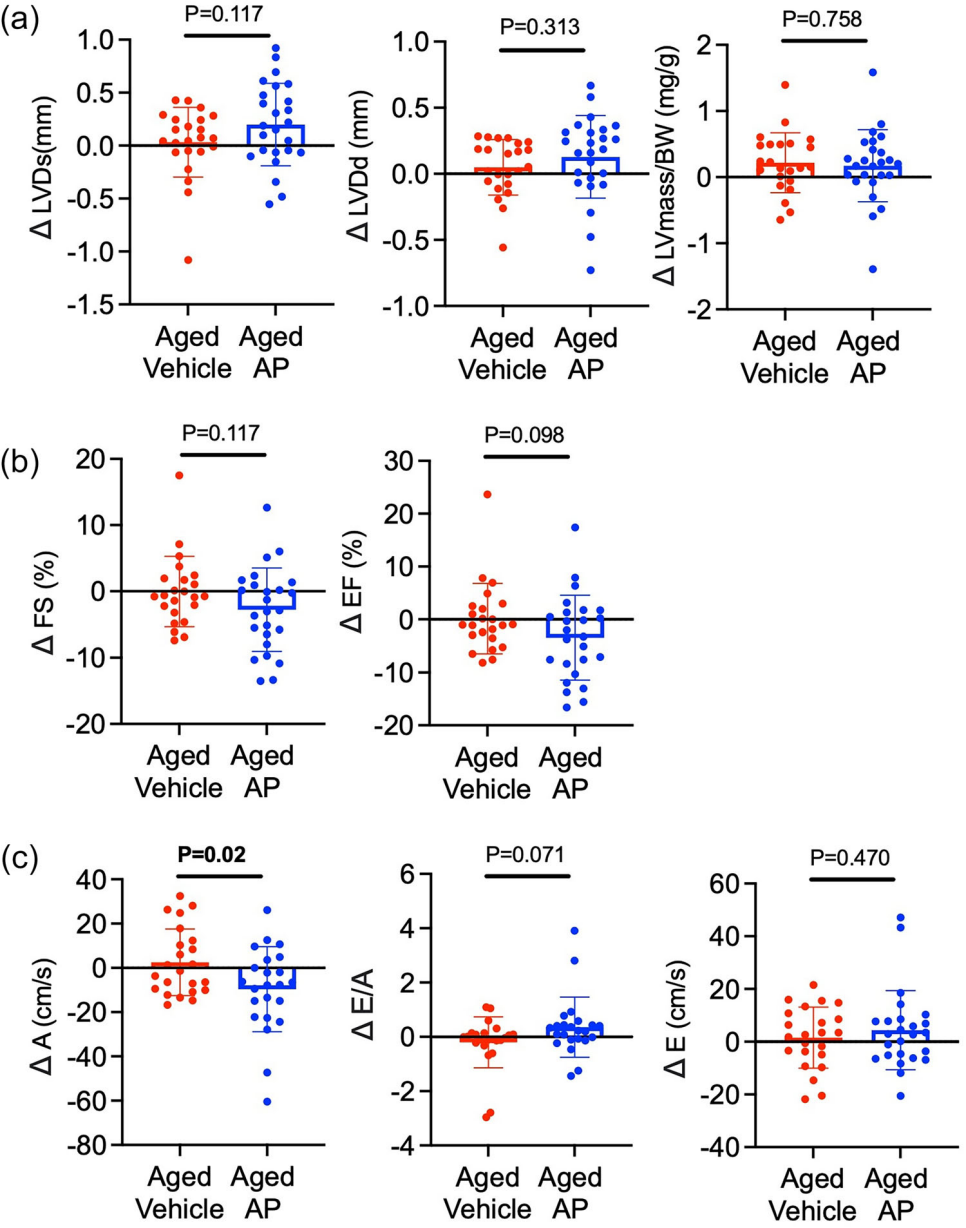
Echocardiographic assessment of cardiac structure and function in aged INK‐ATTAC mice treated with vehicle or AP. The echocardiographic data are reported as changes (Δ) from 12 to 18 months in each group (Aged‐Vehicle, Aged‐AP). (a) Left ventricular (LV) structural parameters: LV dimension at end systole (LVDs); LV dimension at end diastole (LVDd); LV mass/body weight (BW). (b) Systolic function parameters: fractional shortening % (FS%), ejection fraction % (EF%), cardiac output (CO). (c) LV diastolic function parameters: transmitral flow atrial filling (a), transmitral flow early filling (e). Statistics: Unpaired *t*‐test for parametric or Mann‐Whitney test for non‐parametric variables. Each point represents results from one mouse; bars and horizontal lines are means and SDs.

The INK‐ATTAC system selectively targets p16‐positive cells. Therefore, we refer to the targeted population as p16‐positive cells throughout the manuscript. The echocardiographic data in Figure 1 are reported as absolute values as well as changes occurring during the AP‐ or vehicle‐treatment period from the pretreatment 12‐month echocardiogram to the post‐treatment 18‐month study in each group (Vehicle‐treated mice, Aged Vehicle: *n* = 23; AP‐treated mice, Aged AP: *n* = 25). Figure 2 shows changes from 12‐month baseline for a number of other variables. AP prevented the increases in LV mass/LVDd and LVAWT that occurred in the Aged‐Vehicle group (Figure 1b). The LV mass, LVDs, LVDd, and LV mass/BW changes were not significantly different between the two groups (Figures 1d and 2a). Left‐ventricular systolic function (LVFS% and LVEF%) changes were also not statistically different between groups (Figure 2b).

The A‐wave amplitude decreased during treatment in the Aged‐Vehicle group (Figure 2c); the decrease was prevented by AP‐treatment. There were no significant differences in ΔE/A responses over time between the AP versus Vehicle group (Figure 2c).

Unlike echocardiographic indices, which could be repeated non‐invasively before and after AP or vehicle treatment periods, the other variables we analyzed were only available at the end of the study period, so we compared end‐study results in Aged‐AP and Aged‐Vehicle groups to each other and to Young mice. Consistent with an effect on LVH, the mouse heart weight normalized to body weight (HW/BW) (Young mice: *n* = 20; Aged mice: Vehicle‐treated: *n* = 23; AP‐treated: *n* = 25) was significantly greater in the Aged‐Vehicle group compared to the Young group, but unchanged versus Young in the AP group (Figure 1e).

### 3.2 Effect of the clearance of p16‐positive cells on end‐study hemodynamic parameters

**FIGURE 3:**
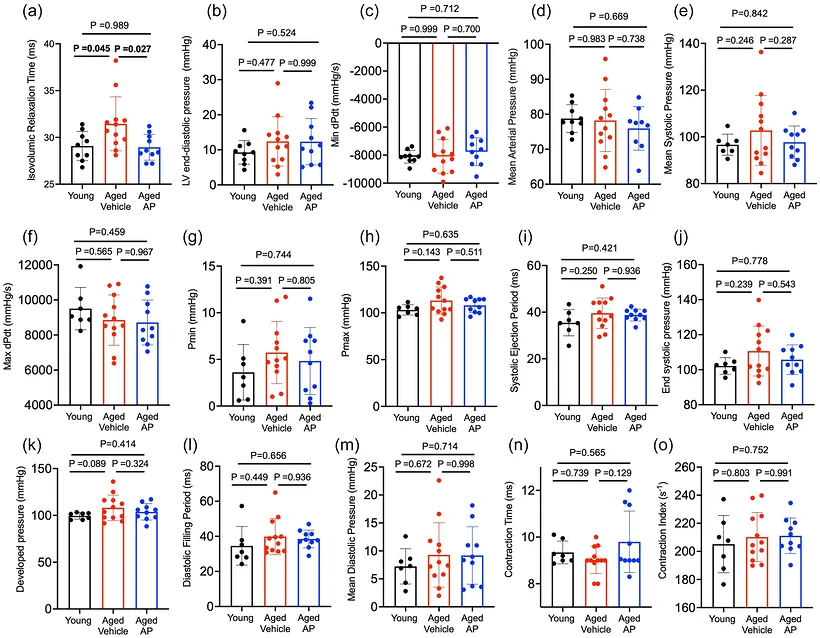
Hemodynamic parameters in Young, Aged‐Vehicle and Aged‐AP INK‐ATTAC. (a) Isovolumic relaxation time. (b) End‐diastolic left ventricular (LV) pressure. (c) Maximum rate of decrease in pressure during relaxation (Min dP/dt). (d) Mean arterial pressure. (e) Mean systolic pressure. (f) Maximum rate of increase in pressure (Max dP/dt). (g) Minimum pressure (Pmin). (h) Maximum pressure (Pmax). (i) Systolic ejection period. (j) End‐systolic pressure. (k) Developed pressure. (l) Diastolic filling period. (m) Mean diastolic pressure. (n) Contraction time. (o) Contraction index (Young mice: *n* = 9; Aged mice: Vehicle‐treated: *n* = 12; AP‐treated: *n* = 10). Statistical analysis for all panels: one‐way ANOVA followed by Tukey's test. Each point represents results from one mouse; bars and horizontal lines are means and SDs.

Hemodynamic studies showed a prolongation in LV isovolumic relaxation time (IVRT), an index of diastolic function, in Aged‐Vehicle group compared to Young mice, which was significantly attenuated in the *p*‐16 cell‐cleared Aged‐AP mice (Figure 3a; Young mice: *n* = 9; Aged mice: Vehicle‐treated: *n* = 12; AP‐treated: *n* = 10), compatible with a role for senescent‐cell accumulation in aging‐related diastolic function changes. There were no significant changes in other hemodynamic parameters of diastolic function (LV end‐diastolic pressure and maximum rate of decrease in pressure during isovolumetric relaxation (min ‐dP/dt) among groups (Figure 3b,c). Mean arterial pressure (Figure 3d) and several LV systolic function parameters were not statistically different among groups (Figure 3e–o).

### 3.3 Clearance of p16‐positive cells with AP in non‐myocyte cardiac cells

**FIGURE 4:**
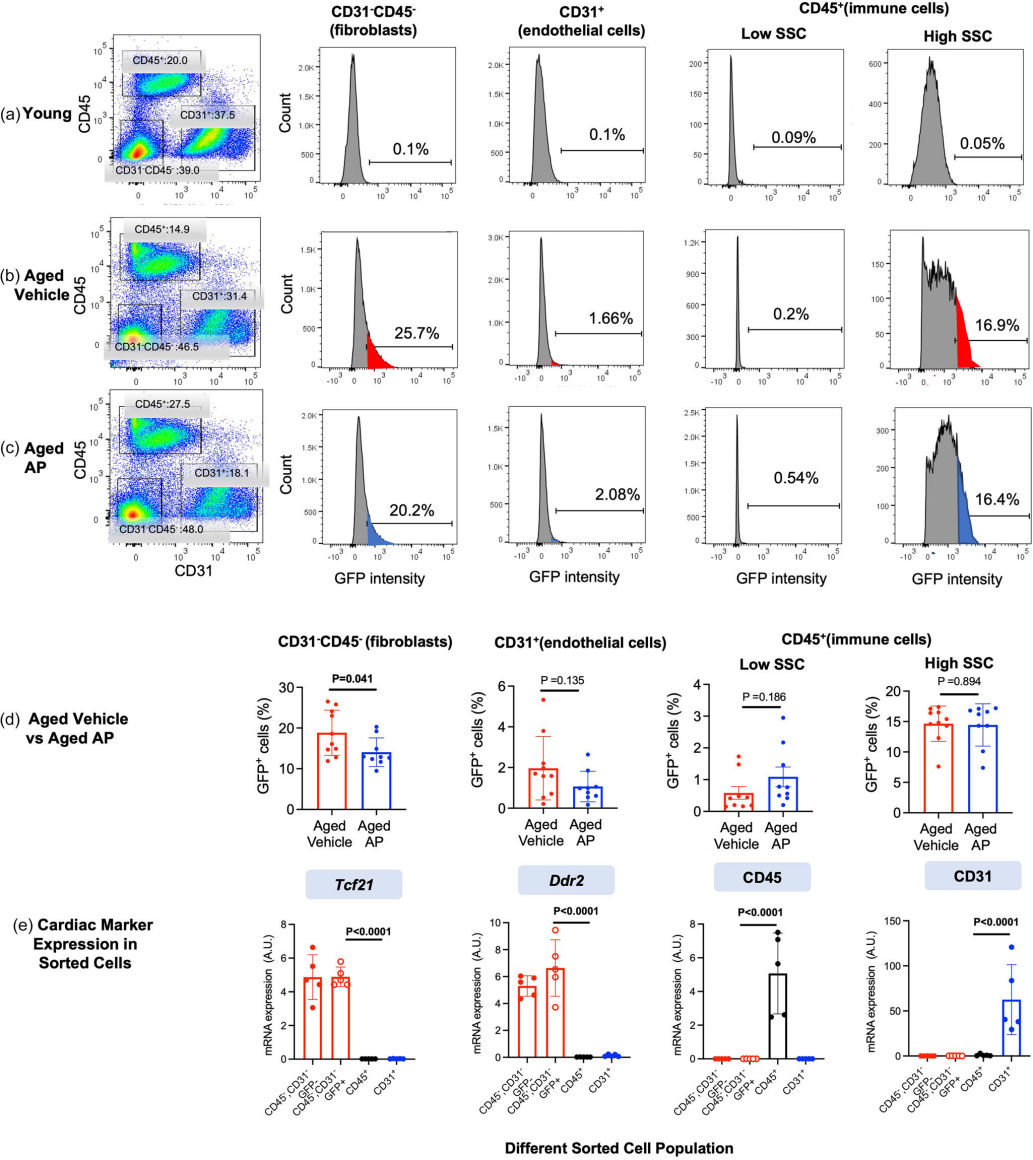
Fluorescence‐activated cell sorting (FACS) for non‐cardiomyocyte cardiac cells in Young, Aged‐Vehicle and Aged‐AP groups. a–c: (a) Young group (b) Aged‐Vehicle group (c) Aged‐AP group. First column: CD31^−^ CD45^−^ (fibroblast) population. Second column: CD31^+^ (endothelial cell) population. Third and fourth columns: CD45^+^ (immune cell) population. (d) Quantification of GFP‐positive (p16‐expressing senescent) cells as a percentage of the total population in each cell type marker for Aged‐Vehicle and Aged‐AP mouse samples. Statistical analysis was performed using an unpaired *t*‐test with a significance level of *P *< 0.05. SSC: Side scatter, indicating granularity of the cells (Aged mice: Vehicle‐treated: *n* = 10; AP‐treated: *n* = 9). (e) Validation of sorted cell population post FACS with qPCR. Fibroblast markers (Tcf21 and Ddr2), immune‐cell marker of (CD45), endothelial‐cell marker (CD31). Statistical analysis was performed using one‐way ANOVA followed by Tukey's test. Ddr2: discoidin domain receptor tyrosine kinase2; Tcf21: transcription factor 21. Each point represents results from one mouse; bars and horizontal lines are means and SD.

The percentage of p16‐positive cells in different non‐myocyte cardiac cell populations (fibroblasts, endothelial cells, and immune cells) was analyzed by FACS (Young mice: *n* = 9; Aged mice: Vehicle‐treated: *n* = 10; AP‐treated: *n* = 9). Young INK‐ATTAC mice were used to determine the GFP background signal (Figure 4a). In all cell populations, the number of p16‐positive cells was increased in aged animals, most significantly in immune cells and fibroblasts (Figure 4b,c). We noted two distinct populations in CD45^+^ cells with different granularity (with high or low side scatter values respectively) and analyzed them separately. Comparison of the GFP signal in each population indicates a statistically significant reduction with AP only in the CD31‐/CD45‐ population (Figure 4d). The FACS experiments were further evaluated with qPCR measurements of the expression of the fibroblast markers transcription factor 21 (*Tcf21*) and collagen‐activated kinase discoidin domain receptor tyrosine kinase2 (*Ddr2*) an immune cell marker (CD45) and an endothelial cell marker (CD31) in the sorted cells (Figure 4e). *Tcf21* and *Ddr2* were strongly expressed in CD45^−^CD31^−^ cells and not at all in CD45^+^ or CD31^+^ cells isolated by FACS, validating their identification as fibroblasts. In contrast, the FACS‐sorted cells expressed CD45 or CD31 exclusively in the cells isolated with the corresponding probe.

**FIGURE 5:**
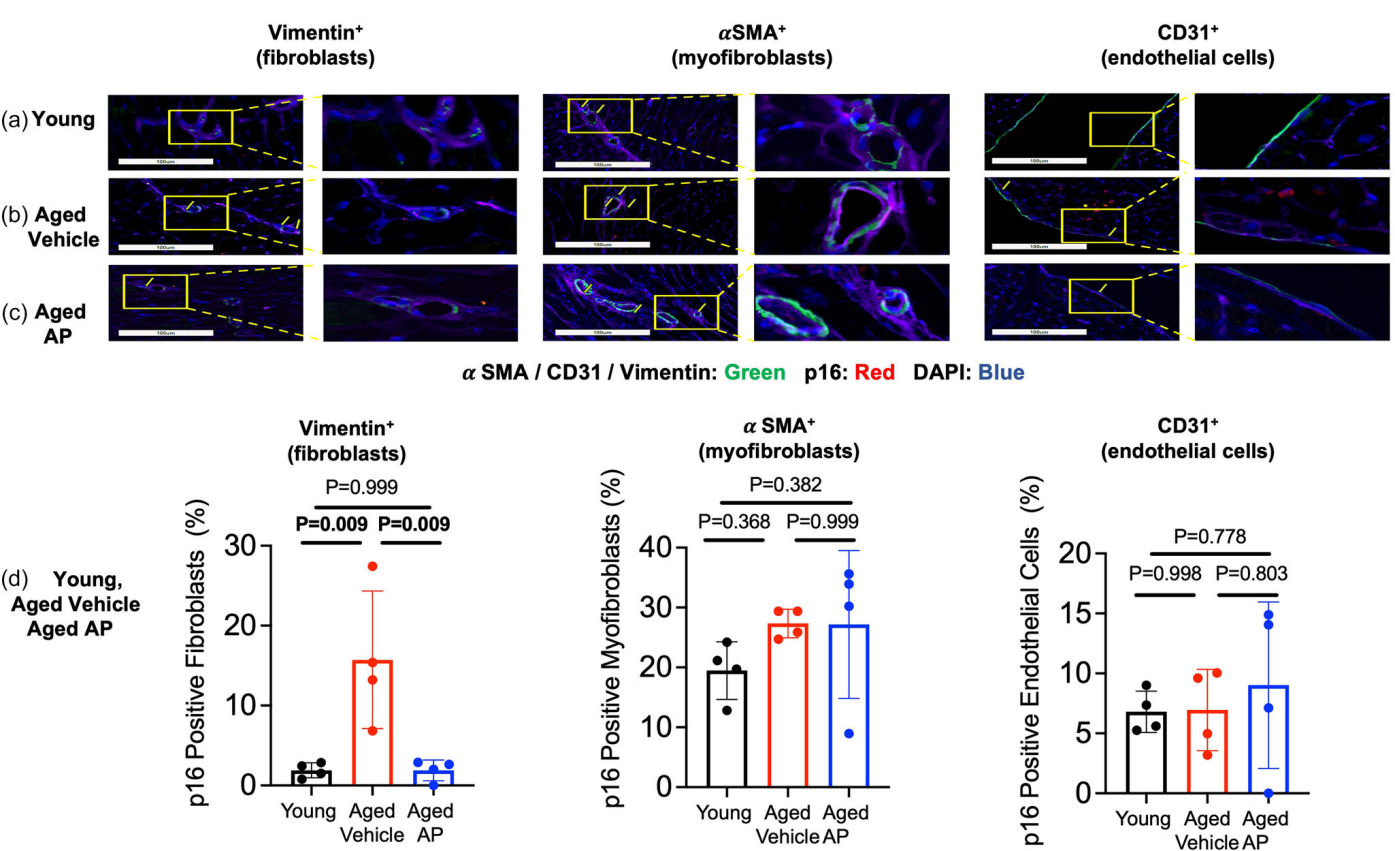
Double immunofluorescence for p16 and cardiac non‐cardiomyocyte cell type markers in left ventricle of Young, Aged‐Vehicle and Aged‐AP groups. (a) Young (b) Aged‐Vehicle (c) Aged‐AP results. For all groups, left column: double staining of p16 and Vimentin (marker of fibroblasts), middle column: double staining of p16 and aSMA (marker of myofibroblasts), right column: double staining of p16 and CD31 (marker of endothelial cells). Each column contains two panels, one (left) at lower magnification and another (to the right) showing magnified views of the area in the yellow box to the left. All panels show cell‐marker staining (green), staining for p16 (red) and 4,6‐diamino‐2‐phenylindole (DAPI, Blue), wheat germ agglutinin (WGA, purple).Yellow arrows point to double‐stained cells (p16 and cardiac cell type marker). The horizontal white lines in the left panels are 100 µm scale markers (Young mice: *n* = 5; Aged mice: Vehicle‐treated: *n* = 5; AP‐treated: *n* = 5). (d) Quantification of the co‐immunostaining data corresponding to the experiments shown above. Statistical analysis for all panels: one‐way ANOVA followed by Tukey's test. Each point represents results from one mouse; bars and horizontal lines are means and SD.

The FACS data were further validated by double immunofluorescence staining of p16 and cardiac cell‐type selective markers (Figure 5) (Young mice: *n* = 4; Aged mice: Vehicle‐treated: *n* = 4; AP‐treated: *n* = 4). AP treatment significantly reduced the percentage of p16‐expressing cells in the vimentin‐expressing population (fibroblasts), while the number of αSMA‐positive cells (myofibroblasts) or CD31‐positive cells (endothelial cells) expressing p16 were unaffected (Figure 5d). α

### 3.4 Effect of p16‐positive cell clearance on cardiomyocytes

**FIGURE 6:**
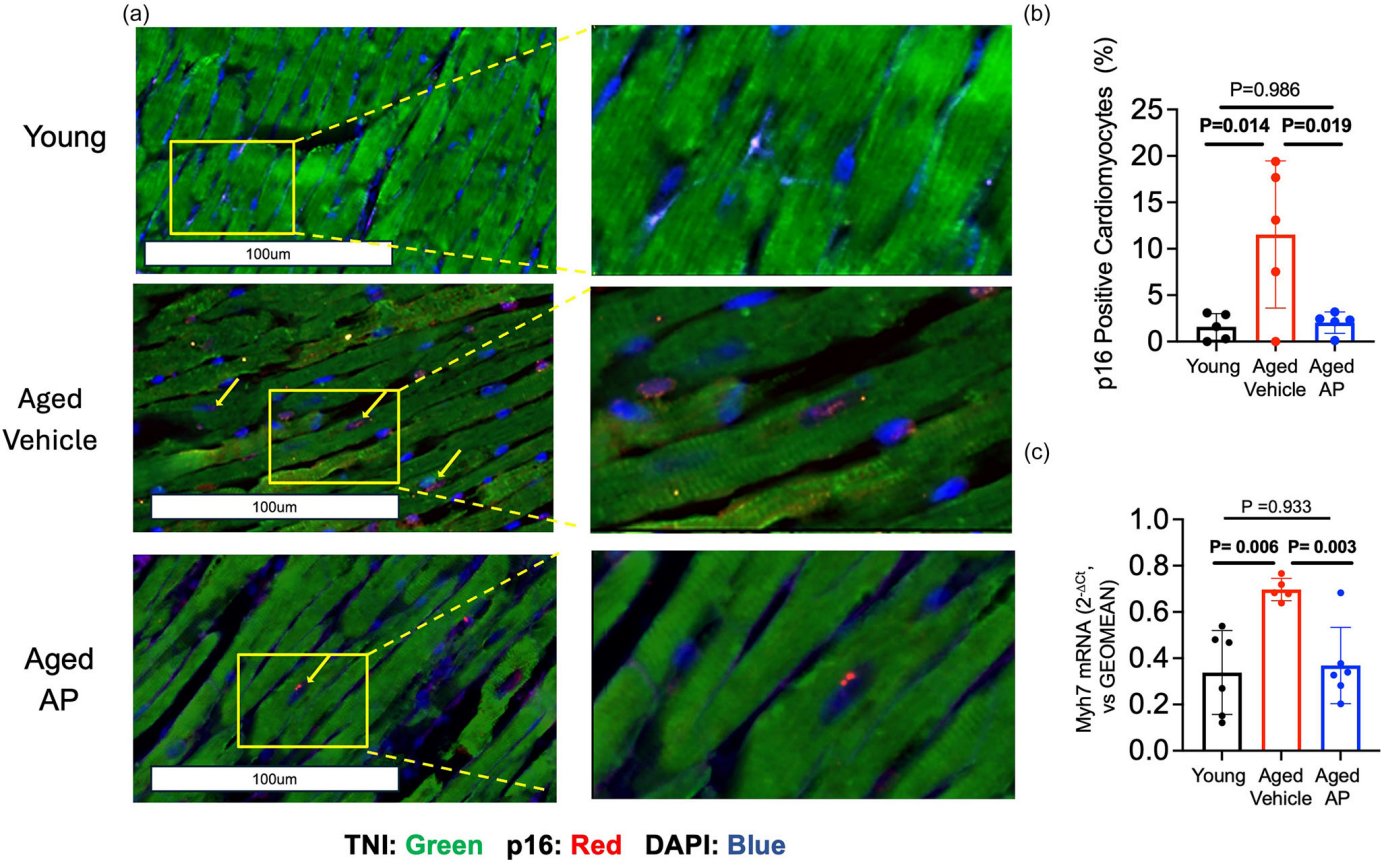
Double immunofluorescence for p16 and cardiomyocyte marker proteins. (a) Heart‐tissue sections stained with senescent‐cell marker p16 (red), nuclear stain 4,6‐diamino‐2‐phenylindole (DAPI; blue), and cardiomyocyte marker, troponin I (green) (Young mice: *n* = 5; Aged mice: Vehicle‐treated: *n* = 5; AP‐treated: *n* = 5). (b) Data are expressed as the percentage of p16^+^ cardiomyocyte nuclei relative to the total number of cardiomyocyte nuclei detected per section.(c) Myosin heavy chain 7 (*Myh7*) gene‐expression. Statistical analysis for all panels: one‐way ANOVA followed by Tukey's test. Each point represents results from one mouse; bars and horizontal lines are means and SD.

**FIGURE A1:**
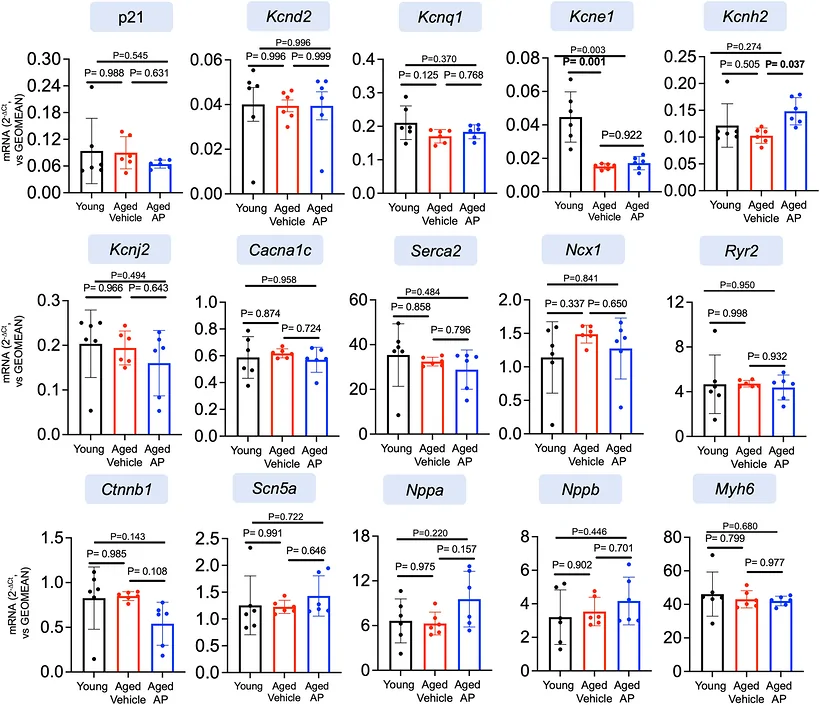
Hypertrophy markers, ion channels and calcium handling proteins gene expression. Statistical analysis for all panels: one‐way ANOVA followed by Tukey's test, significance level *P* < 0.05. (Young mice: *n* = 6; Aged mice:Vehicle‐treated: *n* = 6; AP‐treated: *n* = 6). Each point represents results from one mouse; bars and horizontal lines are means and SD.

Because of the large size of cardiomyocytes, they did not pass through the cell‐filter of the FACS apparatus, and we were unable to separate them from other cells by FACS. Instead, we double‐stained p16 and troponin I (TNI) to identify *p*16‐positive cardiomyocytes on paraffin‐embedded sections (Young mice: *n* = 5; Aged mice: Vehicle‐treated: *n* = 5; AP‐treated: *n* = 5). The percentage of p16‐positive cardiomyocyte nuclei was significantly greater in Aged‐Vehicle versus Young mice, with AP‐treatment reducing the value in Aged‐AP mice to values equivalent to those in Young (Figure 6a,b). We also used purified cardiomyocytes to quantify the expression of genes of interest with qPCR. The mRNA‐expression of myosin heavy chain 7 (*Myh7*), a marker of LVH, was significantly upregulated in the Aged‐Vehicle group compared to the Young, and significantly reduced in the AP‐treated group versus Vehicle (Figure 6c). We also analyzed mRNA‐expression for a variety of ion‐channel, Ca^2+^‐transporter and other cellular markers (p21, *Kcnd2, Kcnq1, Kcne1, Kcnh2, Kcnj2, Cacna1c, Serca2, Ncx1, Ryr2, Ctnnb1, Scn5a, Nppa, Nppb, and Myh6*), finding them not to differ significantly among groups (See Appendix, Figure A1).

### 3.5 Effect of p16‐positive cell clearance on LV fibrosis and conduction velocity

**FIGURE 7:**
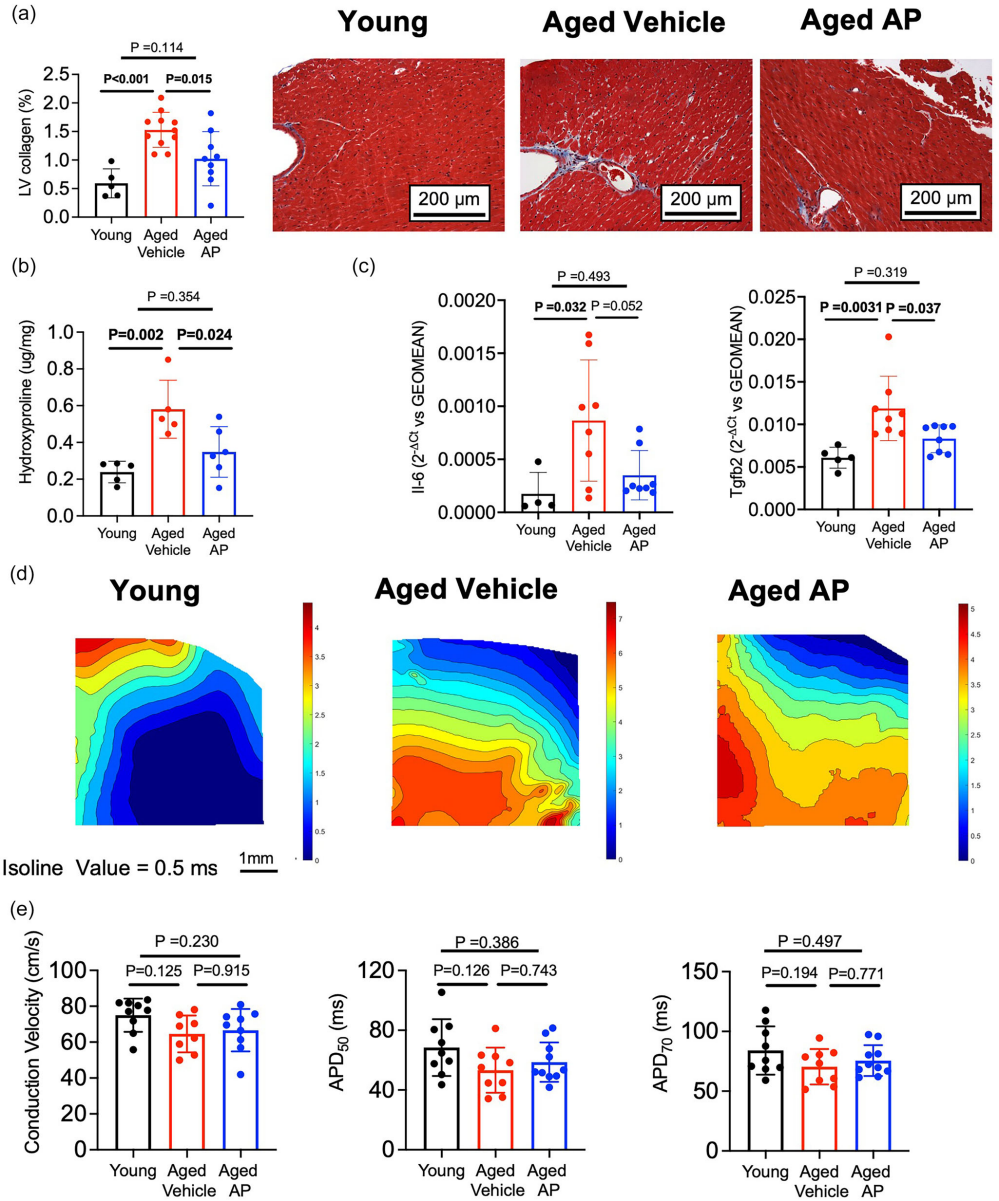
Left‐ventricular fibrosis quantification, profibrotic marker gene expression and optical mapping. (a) Collagen content analyzed with Masson's Trichrome immunostaining (Young mice: *n* = 5; Vehicle‐treated mice: *n* = 11; AP‐treated mice: *n* = 9). (b) Hydroxyproline quantification. (c) Profibrotic marker gene expression. (d) Activation map. (e) Conduction velocity, APD 50, APD 70, APD 90 (Young mice: *n* = 9; Aged mice: Vehicle‐treated: *n* = 10; AP‐treated: *n* = 10). Statistical analysis for all panels: one‐way ANOVA followed by Tukey's test. Each point represents results from one mouse; bars and horizontal lines are means and SD.

The changes in IVRT and diastolic filling velocity are compatible with impaired diastolic function, consistent with the structural and biomarker evidence we obtained for LVH, which increases cardiac stiffness and can impair diastolic function. However, cellular senescence can also be associated with the development of fibrosis, which can equally affect LV compliance (Mehdizadeh et al., 2022). We assessed LV fibrosis by both histological (Masson's Trichrome staining) and biochemical (hydroxyproline assay) approaches. The results were consistent with both methods, suggesting that LV‐collagen content increased in Aged‐Vehicle mice and that this increase was reversed with AP‐treatment (Young mice: *n* = 5; Aged mice: Vehicle‐treated: *n* = 11; AP‐treated: *n* = 9) (Figure 7a,b). The gene‐expression of the profibrotic/SASP markers *Il‐6* and *Tgfβ2* was upregulated in Aged‐Vehicle mice, an effect reversed with AP‐treatment (Young mice: *n* = 4 Aged mice: Vehicle‐treated: *n* = 8; AP‐treated: *n* = 8) (Figure 7c). LV conduction velocity and action potential duration were not different among different groups (Young mice: *n* = 9; Aged mice: Vehicle‐treated: *n* = 10; AP‐treated: *n* = 10) (Figure 7d,e), suggesting that the structural and functional changes we observed were insufficient to alter LV electrophysiological properties.

### 3.6 Paracrine effects of senescent cardiac fibroblasts on healthy cardiomyocytes

**FIGURE 8:**
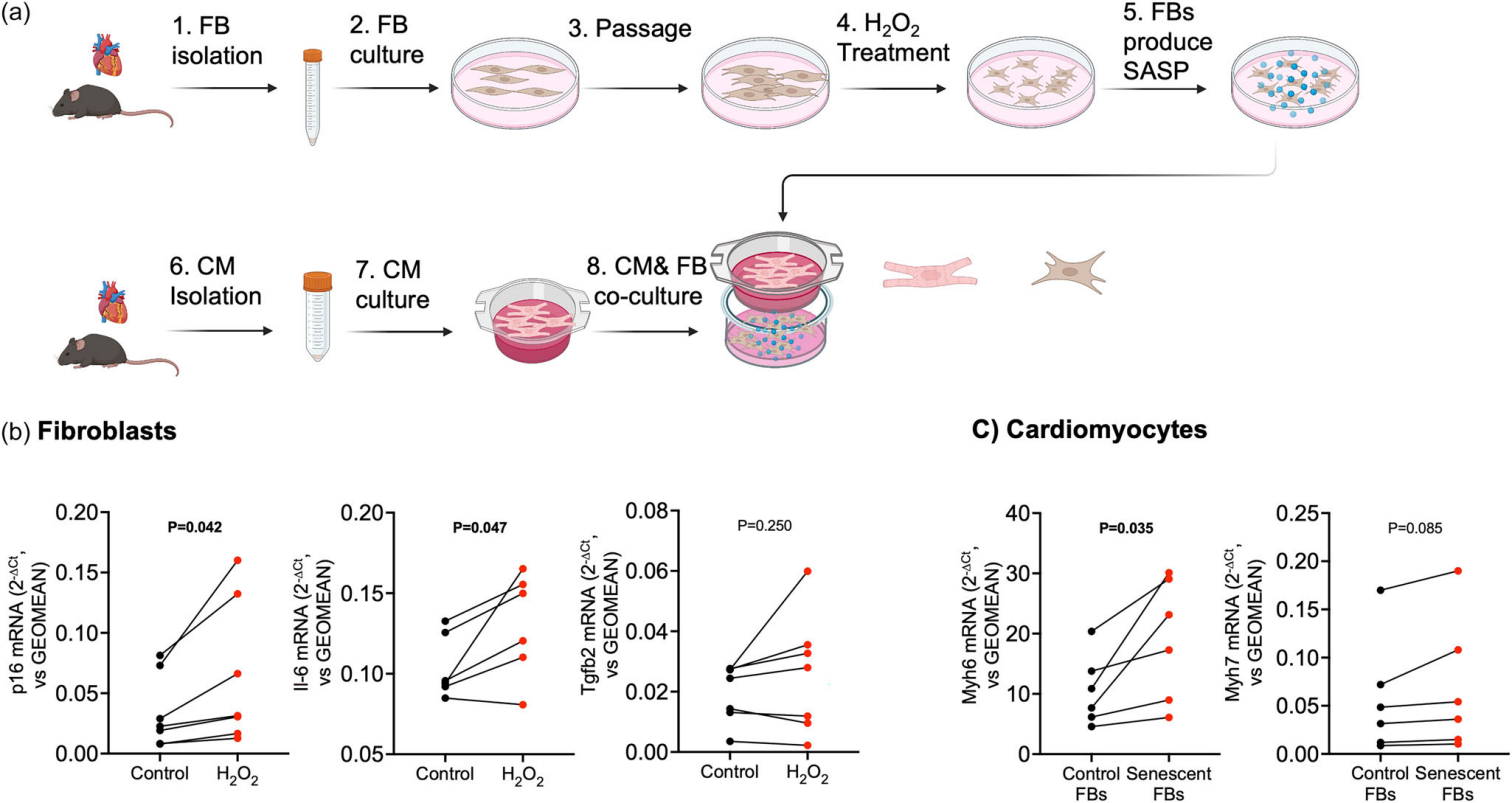
In vitro model of cardiomyocyte exposure to senescent cardiac fibroblast secretome. (a) Study design (b) Senescence and SASP marker gene expression in control fibroblasts and those treated with 200 µM H_2_O_2_ (*n* = 8). (c) Hypertrophy marker gene expression in cardiomyocytes co‐cultured with control versus senescent fibroblasts. Statistical analysis for all panels: unpaired *t*‐test. Each point represents results from one mouse; bars and horizontal lines are means and SD.

Fibroblasts are known to interact with cardiomyocytes via secreted factors, so we wondered whether fibroblast products might be influencing cardiomyocyte changes like hypertrophy. We therefore exposed cultured fibroblasts to hydrogen peroxide to induce cellular senescence (Chen & Ames, 1994; Papaconstantinou, 2019), and cocultured them with cardiomyocytes in chambers that allowed secretome diffusion but blocked cellular migration (Figure 8a). To validate the senescence phenotype in the treated fibroblasts, we measured gene expression of *p16* and SASP markers such as *Il‐6* and *Tgfβ2*. *Il‐6* and *p16* were significantly upregulated in fibroblasts treated with 200 µM H_2_O_2_ (Figure 8b). Next, we cocultured senescent or control fibroblasts with freshly‐isolated cardiomyocytes using Transwell inserts with 0.4 µm pores. After 48 h of coculture, we found that the expression of hypertrophy markers such as *Myh6* and *Myh7* (*n* = 8) was significantly upregulated in the cardiomyocytes exposed to senescent fibroblast secretome compared to control fibroblast secretome (Figure 8c).

## 4 DISCUSSION

In this study, we investigated the role of p16‐positive cells in age‐related cardiac remodeling using the INK‐ATTAC mouse model. We found evidence for the accumulation of p16‐positive cells in the LV of aged mice accompanied by signs of cardiac hypertrophy, fibrosis, and diastolic dysfunction. Clearance of p16‐expressing cells with AP treatment in the INK‐ATTAC mouse model suppressed the indicators of hypertrophy, fibrosis, and diastolic dysfunction, likely via effects on fibroblasts and cardiomyocytes. These results point to a potential role of senescent fibroblasts and cardiomyocytes in age‐related cardiac remodeling. Exposure of healthy cardiomyocytes to diffusible factors from senescent fibroblasts upregulated hypertrophic gene expression in cardiomyocytes, suggesting potential paracrine effects of senescent fibroblast SASP components on cardiomyocytes.

### 4.1 Cellular senescence and age‐related cardiac remodeling

Growing evidence indicates that cellular senescence plays a crucial role in the pathophysiology of age‐related cardiac disease (Evangelou et al., 2023; Gharagozloo et al., 2024; Luan et al., 2024). Clinical and experimental studies have linked cellular senescence, the accumulation of senescent cells, and the production of SASP components to various age‐related cardiac pathologies, including HF, myocardial ischemia and infarction, and cancer chemotherapy‐related cardiotoxicity (Mehdizadeh et al., 2022).

Global elimination of senescent cells in aged mice—through the use of INK‐ATTAC transgenic mice to clear p16‐positive cells or navitoclax treatment—has been shown to reduce fibrosis and cardiomyocyte size without significantly altering LV mass (Anderson et al., 2019). In Baker et al. (2016), vehicle‐treated and AP‐receiving INK‐ATTAC mice showed no significant differences in cardiac rate, LV mass, thickness or diameter, LVEF, or LV fractional shortening (Baker et al., 2016). The changes we noted with AP‐receiving INK‐ATTAC mice in the present study were subtle but consistent, including attenuated increases in LV mass normalized to diameter and LV anterior wall‐thickness, reduced heart weight/body weight increase, prevention of increased IVRT, and reduced LV collagen accumulation.

A major limitation of the current literature on cardiac senescence is that most studies report tissue‐level changes without defining the roles of senescence in specific cardiac cell types. It remains unclear which cardiac cell‐types are most susceptible to senescence in aging‐related disease and which cell populations drive pathological remodeling. The cell‐specific mechanisms underlying the effects of senescence on age‐related cardiac remodeling are often overlooked, highlighting the need for more targeted investigations (Gharagozloo et al., 2024; Luan et al., 2024; Mehdizadeh et al., 2022; Zhai & Sadoshima, 2024). Here, we combined FACS and immunostaining to identify p16‐positive cardiomyocytes and fibroblasts, linking p16‐positive cell clearance to specific cellular targets.

In our previous study, we provided evidence for the accumulation of senescent cells in the atria of aged rats and in the atria of rats with LV dysfunction due to myocardial infarction (MI), where this accumulation was associated with the development of a pathological atrial fibrillation (AF) substrate. Furthermore, we demonstrated that the selective clearance of senescent myofibroblasts and endothelial cells by a combination of dasatinib and quercetin as senolytic therapy prevented atrial fibrosis, and mitigated AF substrate formation in post‐MI rats (Mehdizadeh et al., 2024). A recent randomized clinical study of our group demonstrated that quercetin alone induced senolysis, and reduced post‐cardiac surgery *de novo* AF by more than 75% in patients with symptomatic CAD, further strengthening the clinical relevance of targeting senescent cells (Mury et al., 2025).

In this study, we provide evidence for the potential role of cellular senescence in age‐related cardiac remodeling. To comprehensively assess cardiac function, we implemented multiple approaches, including echocardiography, hemodynamic measurements, ex vivo optical mapping, along with FACS and immunostaining. We demonstrate that clearing p16‐positive cells alleviates structural and functional signs of diastolic dysfunction. Furthermore, our findings suggest that this effect is potentially mediated through the selective elimination of *p*‐16 positive senescent cardiomyocytes and fibroblasts.

### 4.2 Potential mediators of the induction by cellular senescence of age‐related diastolic dysfunction

#### 4.2.1 Cardiac hypertrophy

One potential mechanism through which cellular senescence induces diastolic dysfunction is cardiac hypertrophy, a recognized feature of cardiac aging (Heinzel et al., 2015; Mehdizadeh et al., 2022). Anderson et al. showed that ROS and mitochondrial dysfunction led to telomere‐associated DNA damage, followed by the induction of senescence and hypertrophy (Anderson et al., 2019). In the same study, senescent cell‐clearance in aged mice with navitoclax, or in the INK‐ATTAC p16‐positive cell‐clearance mouse model, reduced the size of cardiomyocytes with no changes in LV mass or cardiac function. The senescent cell‐types cleared by senolytic treatment in their mouse model were not addressed (Anderson et al., 2019).

Here, we found that the clearance of p16‐positive cells in INK‐ATTAC mice prevents cardiac hypertrophy, evident in the reduction of LV mass, LVAWT, and *Myh7* gene expression. Our immunofluorescence results showed a significant reduction in the density of p16‐positive cardiomyocytes with AP‐treatment. Among nonmyocyte cardiac cells, we noted in our FACS experiment that the beneficial effect of AP‐treatment is associated with clearance of p16‐positive fibroblasts. Furthermore, we found that senescent‐fibroblast medium induces cardiomyocyte hypertrophy, suggesting a potential role of paracrine influences from senescent fibroblasts in the production of LVH. Our findings point to the need for careful analysis of heterocellular interactions in the mediation of senescence‐related changes. Further work is needed to identify the diffusible mediator(s) produced by senescent fibroblasts that cause cardiomyocytes to hypertrophy.

Because cardiomyocytes are largely post‐mitotic and the adult heart has limited regenerative capacity, elimination of senescent cardiomyocytes could theoretically have detrimental effects if excessive cell loss occurs. However, in the present study p16‐positive clearance was associated with improved cardiac structure and function in aged mice, suggesting that removal of dysfunctional senescent cells may be beneficial in this context, although the long‐term consequences of senescent cell elimination in post‐mitotic tissues remain to be fully determined (Owens et al., 2021).

#### 4.2.2 Cardiac fibrosis

Another mechanism through which cellular senescence causes diastolic dysfunction might be the promotion of cardiac fibrosis (Abdelfatah et al., 2019; Gutierrez‐Fernandez et al., 2015; Mehdizadeh et al., 2022). Myocardial tissue fibrosis leads to greater cardiac stiffness and impaired relaxation (Peikert et al., 2026). Studies in animal models and in human hearts have confirmed the accumulation of senescent fibroblasts in fibrotic areas of the heart during aging and in association with cardiac disease. Here, we demonstrated that clearance of p16‐overexpressing cells reduces age‐related accumulation of fibrous tissue, and for the first time, to our knowledge, obtained evidence for direct targeting of fibroblasts by the senescent cell‐directed intervention that we used. Baker et al. (2016) did not report changes in myocardial collagen accumulation with the same intervention, while Anderson et al. (2109) noted significant fibrosis attenuation. In the present study, we combined histological analysis of collagen deposition with a quantitative hydroxyproline assay, allowing a more sensitive and comprehensive evaluation of fibrotic remodeling. Fibroblast p16‐positive cell changes were evaluated by both FACS and double‐immunostaining histological analysis, with clearance of p16‐positive fibroblasts by AP treatment indicated by both methods.

#### 4.2.3 Diastolic dysfunction and aging‐related HF

Elderly individuals are particularly prone to HF with preserved ejection fraction (HFpEF) (Gharagozloo et al., 2024). Diastolic dysfunction is an important contributor to HFpEF and is known to become more likely with aging (Anderson et al., 2022; Gharagozloo et al., 2024). Our mice did not show overt HFpEF, but did show that p16‐positive cell clearance reverses prolonged isovolumic relaxation time in elderly mice, a characteristic sign of diastolic dysfunction, in combination with the suppression of cardiac hypertrophy and fibrosis, typical causes of diastolic dysfunction (Anderson et al., 2022). Diastolic function was not specifically assessed in Baker et al. (2016) or Anderson et al. (2019), which primarily focused on global systolic indices such as ejection fraction and fractional shortening. In contrast, our study combined echocardiography with invasive pressure–hemodynamic measurements, enabling detection of changes in isovolumic relaxation time and providing a more sensitive assessment of diastolic function. In our study, p16‐positive cell clearance did not improve ejection fraction or fractional shortening, while a small, statistically non‐significant quantitative decrease was observed (EF *P *= 0.098; FS *P* = 0.117). This observation is consistent with an HFpEF‐like phenotypes, in which systolic function is often preserved while diastolic dysfunction, usually due to increased myocardial stiffness, is often the dominant driver of cardiac dysfunction. Nevertheless, we cannot exclude a very small but real decrease in systolic function, below our level of detection, resulting from p16‐positive cell clearance.

### 4.3 Cardiomyocyte‐fibroblast paracrine interactions in cardiac hypertrophy

It is well‐recognized that different cardiac cell‐types including both cardiomyocytes and nonmyocyte cells like fibroblasts, endothelial cells, and immune cells, actively communicate with each other to modulate each other's properties and functions (Bageghni et al., 2018; Fujiu & Nagai, 2014; Kamo et al., 2015). Recent research has pointed to a role for fibroblasts in inducing cardiomyocyte hypertrophy through paracrine factors like fibroblast growth factor 2 (FGF‐2), transforming growth factor beta‐1 (TGFβ1), and insulin like growth factor 1 (IGF‐1) (Bageghni et al., 2018; Fujiu & Nagai, 2014). Here, we show for the first time that the exposure of healthy cardiomyocytes to medium from senescent fibroblasts increases the gene‐expression of hypertrophic markers in cardiomyocytes. This paracrine mechanism was not addressed in Baker et al. (2016) or Anderson et al. (2019) and provides a potential explanation for how senescent fibroblasts might contribute to cardiomyocyte hypertrophy and cardiac remodeling.

### 4.4 Potential limitations

The INK‐ATTAC mouse model has several considerable advantages: the model is specific, it has minimal off‐target effects, and it has been used in prior studies of cellular senescence (Anderson et al., 2019; Baker et al., 2016; Khosla et al., 2020; Mehdizadeh et al., 2022). However, the model has the weakness of inducing apoptosis specifically in p16‐positive cells. While p16 is among the most important senescence markers, not all senescent cells express p16. Thus, not all senescent cells are cleared using this system. Overall, p21 expression at the tissue level was not significantly different in our study between treated and vehicle groups at the mRNA level; however, bulk measurements may not capture cell‐specific senescence signals. Cellular‐level evaluation was limited: in FACS, identification of p16^+^ cells via the GFP reporter precluded simultaneous assessment of p21, and in immunofluorescence, the available p21 antibody did not yield a reliable signal despite optimization attempts. Furthermore, the limited screening panel that we were able to use for senescent cells is insufficient to provide a full test for senescence. We did note decreased expression of the SASP‐markers Il6 and TGFβ2 with p16‐positive cell clearance, consistent with senescent‐cell removal. While we focused on TGFβ2 based on prior work, TGFβ1 was not assessed. Thus, we cannot exclude contributions from non–p16^+^ senescent populations or additional profibrotic mediators. The limited panel of senescence indicators that we could use (because of limited tissue availability) must be considered in interpreting our findings.

Another limitation of this study is that senescent cell clearance was initiated during mid‐to‐late life (12–18 months) and maintained for 6 months, rather than extending intervention into more advanced ages when the senescence burden is higher. As such, the effects of later or longer interventions (e.g., for 9–12 months) further into the period of natural senescence remain unclear and warrant further investigation. Because the INK‐ATTAC model induces systemic rather than tissue‐specific clearance of p16^+^ cells, we cannot exclude a role of indirect systemic and vascular mechanisms (like clearance of senescent cells in vascular compartments known to contribute to age‐related dysfunction) in the observed improvements in cardiac structure and function, in addition to the direct effects on the myocardium that we showed.

In our FACS experiments, we quantified CD31/CD45 double‐negative cells as fibroblasts, since there is no single unique common surface marker for fibroblasts and previous investigators have considered CD31/CD45 double‐negative cells likely to be fibroblasts (Ali et al., 2014; Stellato et al., 2019). Furthermore, qPCR of characteristic fibroblast‐selective markers (*Ddr2* and *Tcf21*) showed enriched expression in the double‐negative population, pointing to a significant contribution of fibroblasts. In addition, our co‐immunostaining experiments confirmed the increased presence of vimentin‐positive, p16‐positive cells in aged vehicle‐treated hearts, which were cleared in the AP‐treated mice (Figure 5d). We were unable to directly measure the size of the cocultured CMs because, after 48 h of co‐culture with fibroblast medium, they became too fragile and detached from the surface, making them unsuitable for immunostaining. Nevertheless, they clearly overexpressed hypertrophic markers. It would have been of interest to quantify a larger number of senescence markers from our tissue samples; however, because of the limited RNA yield from isolated cardiomyocytes, largely resulting from reduced cell viability after 48 h in culture, we were only able to analyze a small number of selected genes. It is also important to note that because only male mice were included, potential sex‐related differences in the observed effects could not be evaluated.

## 5 CONCLUSIONS

In the present study, we show that the clearance of p16‐positive fibroblasts and cardiomyocytes in aged mice reduces cardiac hypertrophy and fibrosis, and improves the LV isovolumic relaxation time. These findings point to a potential role for cardiac cell senescence in aging‐related cardiac hypertrophy, fibrosis, and diastolic dysfunction. These insights may help in the development of innovative therapies for age‐related cardiac disease, particularly HF.

## AUTHOR CONTRIBUTIONS

Mozhdeh Mehdizadeh designed the study with Stanley Nattel, performed most of the experiments, analyzed the data, and drafted the manuscript. Martin Mackasey performed and analyzed the optical mapping experiments and assisted with cardiac cell isolation. Kimia Gharagozloo conducted the in vitro study. Martin Aguilar contributed to data interpretation and analysis. Patrice Naud conducted the qPCR experiments. Nhung Vuong‐Robillard analyzed the immunofluorescence data. Eric Thorin and Gerardo Ferbeyre assisted with study design and data interpretation. Jean‐Claude Tardif supervised the echocardiographic studies and the blinded echocardiographic analyses. Allan Ochs contributed to the immunofluorescence and fibrosis experiments. Martin G. Sirois and Jean‐François Tanguay co‐supervised the histological work and the blinded histological analyses. Stanley Nattel supervised all phases of the study, worked with Mozhdeh Mehdizadeh to design the experiments and analysis, obtained funding for the work, and provided guidance and editing for manuscript preparation.

## CONFLICT OF INTEREST

None declared.

## GENERATIVE AI STATEMENT

The authors declare that no generative artificial intelligence (AI) tools were used at any stage in the preparation of this manuscript.

## Data Availability

All raw data are available from the author on reasonable request.
